# Putting pressure on aromaticity along with *in situ* experimental electron density of a molecular crystal

**DOI:** 10.1038/ncomms10901

**Published:** 2016-03-16

**Authors:** Nicola Casati, Annette Kleppe, Andrew P. Jephcoat, Piero Macchi

**Affiliations:** 1 Paul Scherrer Institute, WLGA/229, CH-5232 Villigen, Switzerland; 2 Diamond light source Ltd., Harwell Science and innovation Campus, Didcot OX110DE, UK; 3 Institute for Study of the Earth's interior, Okayama University, Yamada 827, Misasa, Tottori 682-0193, Japan; 4 Department of Chemistry and Biochemistry, University of Bern, Freiestrasse 3, Bern CH-3012, Switzerland

## Abstract

When pressure is applied, the molecules inside a crystal undergo significant changes of their stereoelectronic properties. The most interesting are those enhancing the reactivity of systems that would be otherwise rather inert at ambient conditions. Before a reaction can occur, however, a molecule must be activated, which means destabilized. In aromatic compounds, molecular stability originates from the resonance between two electronic configurations. Here we show how the resonance energy can be decreased in molecular crystals on application of pressure. The focus is on *syn*-1,6:8,13-Biscarbonyl[14]annulene, an aromatic compound at ambient conditions that gradually localizes one of the resonant configurations on compression. This phenomenon is evident from the molecular geometries measured at several pressures and from the experimentally determined electron density distribution at 7.7 GPa; the observations presented in this work are validated by periodic DFT calculations.

## Abstract (web summary)

Aromatic compounds display enhanced stability due to delocalized π-bonding. Here, the authors study an aromatic, molecular crystal under pressure using x-ray diffraction and calculations, and observe a reduction in π-bond delocalization under increasing pressure.

## Graphical abstract

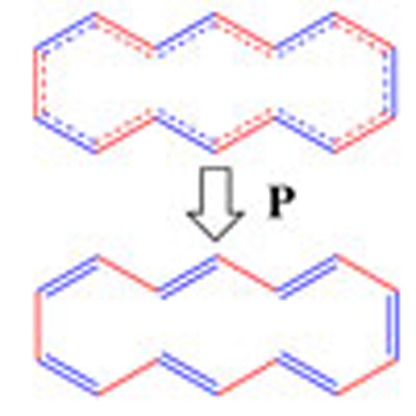

One of the most important and famous classes of chemical compounds is that of aromatic molecules. According to Hückel^1^,^2^, the conjugation of an odd number of electron pairs in a ring is stabilized by the resonance between two equivalent electronic configurations. The implications for the chemistry of these compounds are enormous: the extra stability of aromatic hydrocarbons implies more severe conditions to induce reactions, compared with non-conjugated poly-olefins. Aromaticity affects also the structure of a molecule and its response to external magnetic fields, in particular, the nuclear resonant frequency. In general, a planar geometry formed by equally distant C atoms, an induced diamagnetic current in the ring and a scarce tendency to react are the main clues of aromaticity^3^. Nevertheless, an unbiased and universal criterion for quantifying aromaticity remains elusive. Over the years, chemists have challenged the very concept of aromaticity at times, synthesizing ever more exotic molecular systems and using different investigating methods (diffraction, NMR, reactivity tests, molecular orbital calculations and so on), with the aim of finding universal criteria based on structural, energetic and magnetic parameters^4^,^5^,^6^,^7^,^8^. Insight often came by comparing different molecular species, mimicking a continuous variation of the more relevant parameters, to solve the aromatic riddle^3^,^9^. In this work, instead, we adopt a different strategy to investigate aromaticity, which is probing its variation in a single species, while modifying continuously the molecular geometry through compression.

Reducing the aromaticity of a species requires significant external energy, to stabilize one of the two electronic configurations over the other, breaking the resonance. Typically, chemists make use of heat, light or electrochemical potential to attack an aromatic molecule. An alternative source of external energy is pressure, which rises the internal energy and the enthalpy of a system. This may perturb the molecular conformation and/or the electronic state stable at the ambient conditions, in favour of an otherwise inaccessible configuration.

Recent research in high-pressure (HP) solid-state chemistry led to the discovery of very peculiar phenomena, such as the polymerization of molecules like N_2_ (ref. 10), CO (ref. 11) and CO_2_ (ref. 12), or the transformation of metals into non-metals, for example, Na (ref. 13). Studies on organic crystals are less abundant and have appeared only more recently^14^,^15^. Benzene—that is, the prototype of aromaticity—is an exception, because the first, seminal investigations of its HP forms date back to 1960s (ref. 16). While its phase diagram remains controversial, there is consensus on the occurrence of an irreversible polymerization above 24 GPa, but the structure of this phase is known only from theoretical predictions^17^, without experimental confirmation yet. Ciabini *et al*.^18^ estimated that below a critical intermolecular distance (C–C≈2.6 Å) lattice phonons are able to bring atoms of neighbouring molecules sufficiently close to induce an intermolecular addition reaction, which eventually leads to one or more polymeric products^19^. On crystals of s-triazine, a progressive destabilization of the π bonding orbitals has been reported, based on two-photon induced fluorescence^20^, and the enhanced reactivity of the species was ascribed to this process. In both cases, however, details are missing for the pressure-induced distortions of the molecular geometry that could favour reactivity. In fact, the HP crystalline phases of benzene that anticipate the polymerization^21^ are not known with sufficient precision to enable fine speculations and the high-pressure structures of triazine are poorly characterized. It is also worth noting that most of related studies are conducted in non-hydrostatic conditions.

To obtain relevant information on the aromaticity of species under pressure, the structural, electronic and energetic changes should be monitored. The elective method for investigating molecular geometries is single crystal X-ray diffraction, which maps the electron density (ED, *ρ*(**r**)) distribution in a crystal. Measurements of particular accuracy and completeness are able to reveal not only the maxima of *ρ*(**r**) (coinciding with the nuclear sites) but also the smaller fraction of electrons present in between the atoms and responsible of the chemical bonding^22^. The ED mapping from X-ray diffraction is nowadays a well-established technique, but it requires quenching the atomic motion at low temperature for a sufficient deconvolution of the ED from the atomic displacements. In addition, a more sophisticated modelling based on atomic multipolar expansion^23^ is necessary to extract the desired information from the diffraction data. In this respect, benzene may not be the perfect test case, because its solidification must occur in the HP apparatus and the crystal sample cannot be of the highest quality. Experimental ED determinations of molecular crystals at HP are not known so far. A few examples reported ED maps of simple inorganic compounds^24^ or pure elements^25^, obtained by maximum entropy method. Models using multipoles restricted to theoretical values have been tested against X-ray diffraction data for propionamide^26^ and piperazinium hydrogen oxalate^27^, but no full refinement was so far reported.

The main obstacles to ED mapping from HP X-ray diffraction are due to the pressure apparatus (the Diamond Anvil Cell, DAC), which reduces drastically the resolution, completeness and quality of available data^28^. However, such pitfalls may be overcome by a combination of higher pressure (which significantly attenuates thermal motion even at ambient temperature), modern synchrotron sources (easily providing high-intensity and very short wavelength radiation) and careful experimental strategies; these are discussed in details below.

A complementary approach to study the HP forms of molecules in crystals is first principle calculations, in particular density functional theory (DFT) with periodic boundary conditions^29^. This not only allows to validate the experimental observations and predict the occurrence of new phases, but also to calculate quantities otherwise not available or too difficult to measure, such as electron correlation, current density and electronic energy of a system. Moreover, a theoretical analysis enables extending the pressure range achievable with experiments.

In the following, we report on the experimental and theoretical investigations of an aromatic molecule in its crystal form and we analyse how its aromatic character is reduced by the pressure-induced modifications of the molecular geometry.

## Results

### HP single crystal X-ray diffraction

**Figure 1:**
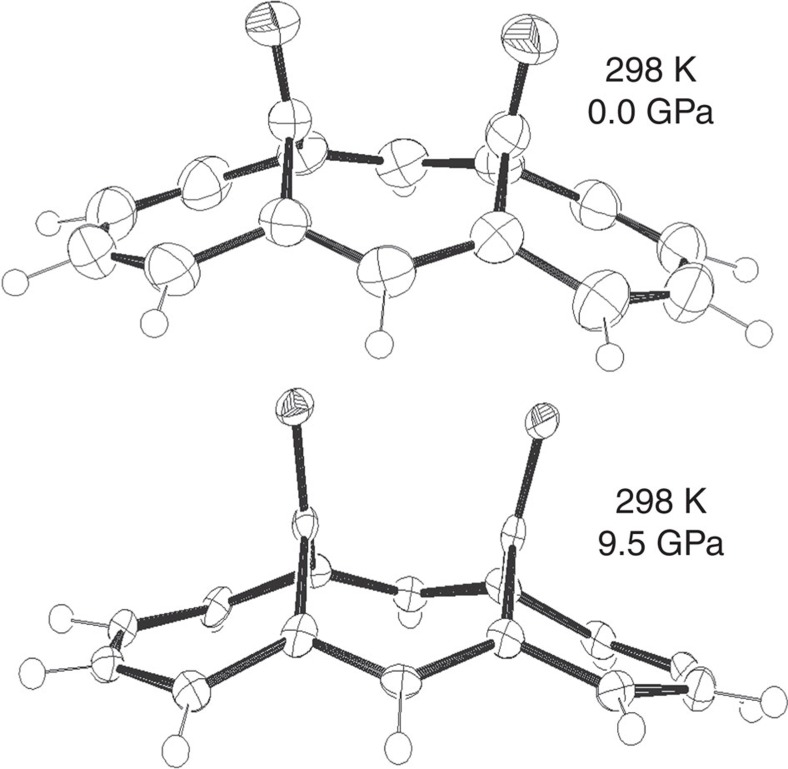
BCA molecule. The molecular geometry and atomic displacement parameters of BCA at ambient pressure and at 9.5 GPa.

Our investigation focused on a doubly bridged annulene, namely *syn*-1,6:8,13-Biscarbonyl[14]annulene (BCA), Fig. 1, for which high-quality crystals are available. Initially studied within the debate on aromaticity of annulenes^30^, BCA was also the subject of a very detailed ED study at ambient *P* and low *T* (19 K) (ref. 31), which is an excellent benchmark. Due to the strain in the ring, the aromaticity of BCA is quite smaller than for benzene, therefore, a stronger response to perturbation is expected.

Single crystals of BCA were investigated with multi-temperature and multi-pressure X-ray diffraction, to determine experimentally the structural changes on varying the thermodynamic conditions. HP diffraction experiments were carried out at the I15 beamline of Diamond light source using a pinhole defined monochromatic beam with 0.31 Å wavelength and an Atlas CCD detector. Six simple data collections, based on perpendicular scans (*ω* and *ϕ* at *χ*=90) with frontal detector (resulting in a resolution up to 0.8 Å), were performed in the range from 0.0001 to 9.5 GPa. These were intended to determine the main structural changes occurring to the molecule as a function of pressure, up to the hydrostatic limit of the pressure-transmitting medium that we adopted (methanol:ethanol 4:1 mixture; *ca.* 10 GPa). The data collection at 7.7 GPa (experiment EE7741-1) was more extensive, involving several *ω* and *ϕ* scans at four different *χ* positions (0°, 30°, 60° and 90°), using different *θ* positions for the detector, as it was intended to measure with the best accuracy the diffraction intensities and to reach the highest resolution (*d*=0.5 Å). Accuracy here means: (a) high data completeness (that is, the portion of reciprocal space that is measurable), obtained using two crystals in the DAC; (b) redundant measures of the diffracted intensities (with every *ω* and *ϕ* scan re-performed with a 2° offset of *ϕ* and *ω*, respectively) to minimize random errors and therefore enable a multipolar expansion of the ED. The high-energy radiation chosen reduces absorption effects and allows collection of higher-order reflections. A beam smaller than the crystal was selected by a 30-μm pinhole, to probe one crystal at a time and to maximize the sample/diamond diffraction intensity ratio. Periodic and molecular DFT calculations were used to simulate the structures, validate the experimental results, compute the geometry at pressures above the experimentally available range and compute those properties that are not directly accessible through experiments, like the current densities and the electron delocalization indices. Details of all experiments and calculations are in Methods section and in Supplementary Methods.

### Theoretical calculations and molecular geometries

**Figure 2:**
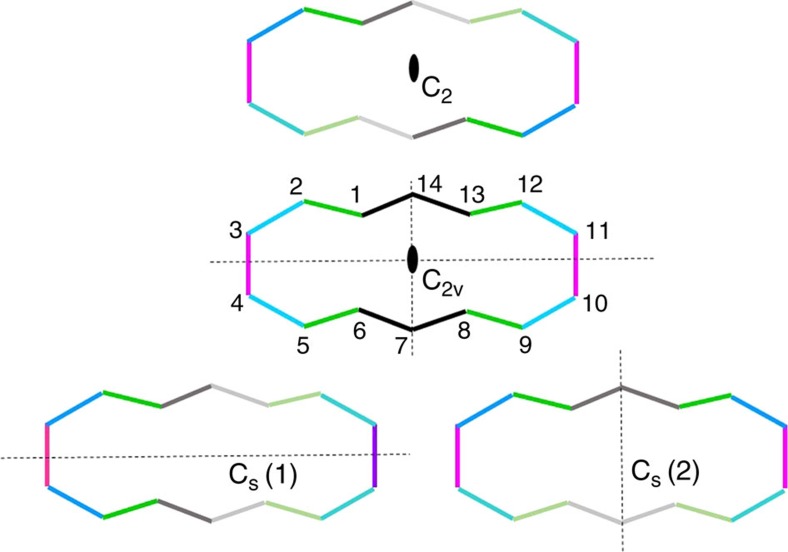
The possible configurations of BCA. BCA symmetries (only the annulene skeleton is drawn): C_2v_ is the most symmetric isomer, stable in the gas phase and very close to the molecule in the crystal at ambient pressure; C_s_(1) is the sub-symmetry which is close to the configuration found at HP; C_2_ and C_s_(2) are the other two possible sub-symmetries. Note that only C_s_(1) implies breaking the resonance between ψ_1_ and ψ_2_.

**Figure 3:**
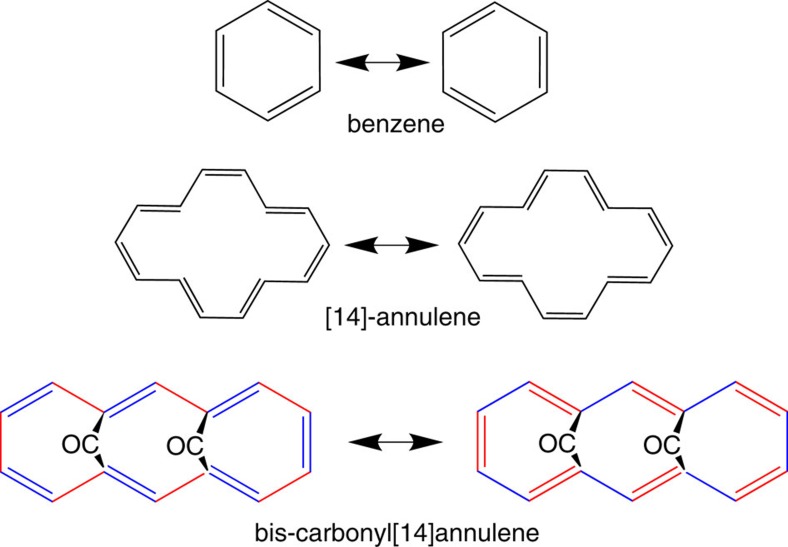
Resonant configurations in aromatic molecules. Skeletal formulae of the resonant configuration in selected aromatic molecules.

With its 14 carbon atom ring, BCA is a 4*n*+2 Hückel system, therefore potentially aromatic. However, the two carbonyl bridges distort the planarity of the ring, reducing the conjugation of C–C bonds and therefore decreasing the aromaticity of the system. The C–C distances are quite important indicators: in an aromatic molecule, the ring skeleton bonds should have homogeneous distances, quite shorter than single bonds but longer than double bonds. In the gas phase of a molecule like benzene, all C–C distances are equal by symmetry (1.389 Å), whereas in BCA the lower molecular symmetry (C_2v_) does not imply equalization of C–C distances and the two bridges produce an heterogeneous distribution in the range 1.37–1.41 Å. Four C–C bonds are symmetry independent, Fig. 2. Gas phase DFT calculations on the isolated molecule (at B3LYP/6–31+G(2d,2p) level) indicate that C1–C14, C2–C3 and all their symmetry equivalents, are shorter whereas C1–C2, C3–C4 and all their symmetry equivalents, are longer. Nevertheless, a C_2v_ symmetry still implies a perfect resonance between the two electronic configurations ψ_1_ and ψ_2_ (Figs 2 and 3). In the crystal phase, the BCA molecule sits on a general position (therefore, without any intramolecular symmetry element), although its geometry remains close to the gas phase C_2v_ isomer, as confirmed by X-ray diffraction. The small root mean square deviation from C_2v_ progressively increases on lowering the temperature (from 0.031 Å at 298 K to 0.036 Å at 19 K). It is worth noting that, even in the absence of carbonyl bridges, a ring strain significantly affects the aromaticity of the ‘parent', unbridged [14]-annulene (Fig. 3), which is in fact not planar^32^.

The aromaticity is not only reflected by the C–C distances, hence by the position of the *ρ*(**r**) maxima, but also by the amount of ED in the chemical bonds, correlated with the bond strength. In this respect, the detailed experimental study by Destro and Merati^31^ on BCA went beyond a routine geometrical analysis, providing also an accurate determination of *ρ*(**r**) at 19 K. At the bond critical points, (that is, saddle points of the three-dimensional ED function, according to Bader's QTAIM^33^), *ρ*(**r**) parallels the bond distances and confirms the perfect resonance between the two electronic configurations. Interestingly, this analysis revealed an unexpected bond critical point interconnecting the two bridging carbons (C15–C16 in Supplementary Fig. 1). Although the ED at the critical point is small, the feature is not anticipated from a simple Lewis structural formula of BCA. Moreover, the C–C distance of 2.593 Å is more than 1 Å longer than a typical covalent bond. A Møller-Plesset^34^ perturbation calculation on the isolated molecule, indicates a small population of two virtual molecular orbitals containing in-phase combinations of the π*-type C=O orbitals. Nevertheless, this interaction is principally of closed-shell type.

As anticipated, the aromaticity also affects the magnetically induced current density in the ring. This modifies substantially the interaction of an external magnetic field with the spin active nuclei, as it can be monitored by nuclear magnetic resonance. However, the atomic chemical shift may depend on the position of the spin active nuclei (most often the protons, revealed by H^1^ NMR). Therefore, geometries of cyclic molecules that differ for conformation may introduce biases because of shielding/deshielding effects not directly related to the aromatic behaviour. For this reason, a nuclear-independent chemical shift (NICS) indicator has been introduced^35^ to provide an unbiased indication of the ring current. NICS is available only from theoretical simulations, because it requires calculating the shielding due to the ring current in the centre of the ring, assuming a virtual atom in that site. The NICS is the negative of the shielding tensor, for sake of consistency with traditional NMR chemical shifts. We calculated a negative NICS at the centre of the BCA ring, which implies the diatropic ring current produced by aromaticity^3^.

**Figure 4:**
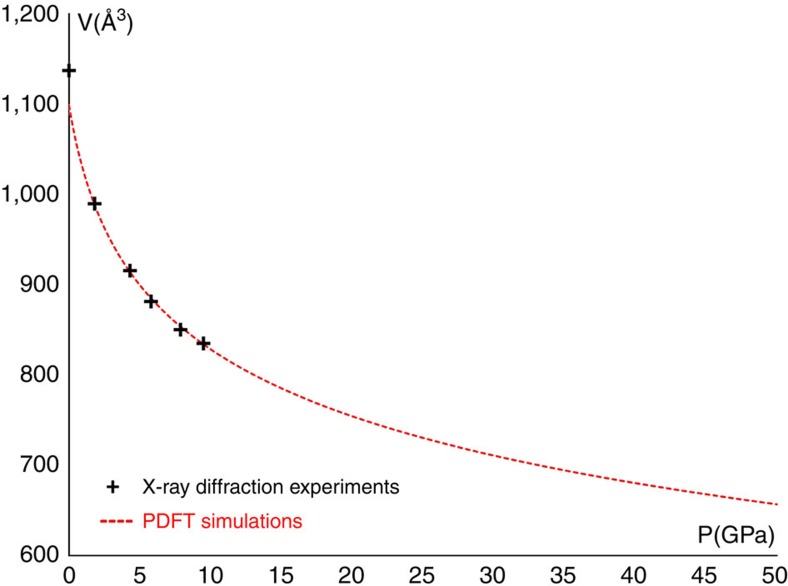
BCA compressibility. The unit cell volume as a function of pressure, from single crystal diffraction experiments and from periodic DFT calculations.

**Figure 5:**
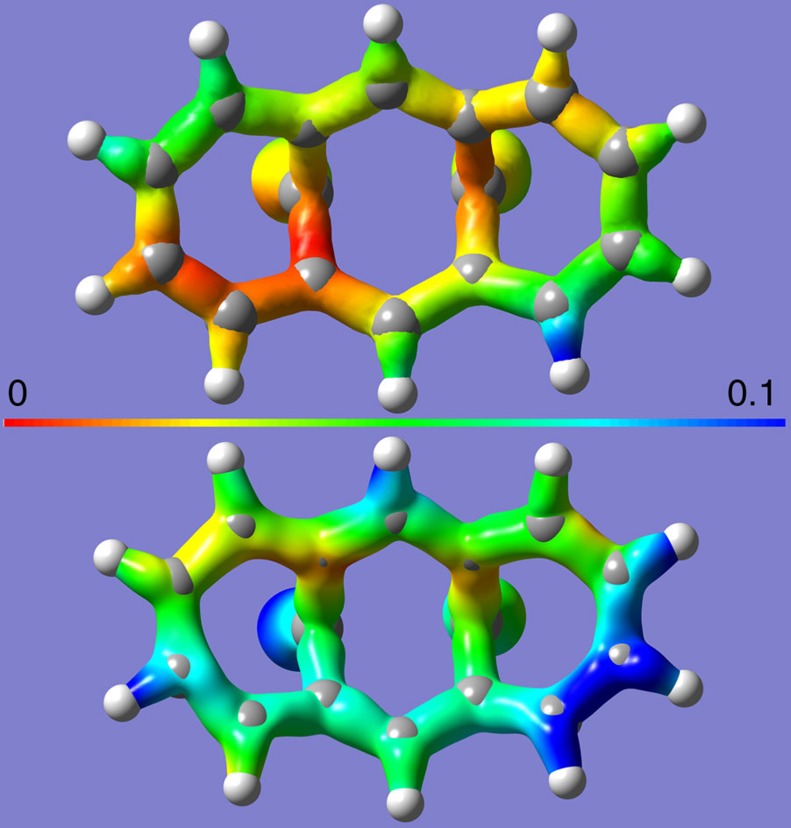
BCA electric field. The magnitude of the electric field acting on a BCA molecule, plotted on a electron density isovalue surface of 0.2 a.u. of BCA at 0.0 GPa (top) and 7.7 GPa (bottom). The colour scale for the electric field is in atomic units (=5.1 × 10^11^ Vm^−1^, blue). a.u., arbitrary units.

As mentioned in the introduction, our goal was observing how all parameters, correlated with aromaticity, vary when the molecule is compressed. The HP experiments revealed a quite large compressibility of the crystal, which exceeds 25% from ambient pressure up to 9.5 GPa, in keeping with theoretical predictions (Fig. 4): the experimental *K*_0_, calculated using a fourth order Birch–Murnagham equation, is only 7.4 GPa (compared, for example, to 37.1 GPa for quartz)^36^. As the crystal shrinks, the electric field experienced by a molecule and generated by all other molecules in the crystal, increases significantly (Fig. 5). Because the molecule sits on a general position, inside the monoclinic unit cell, atoms which would be equivalent under the ideal C_2v_ symmetry of the molecule, experience a different crystal electric field and this asymmetry increases with pressure. Therefore, an external stress gradually, but not uniformly, perturbs all the covalent bonds of the annulene skeleton.

**Figure 6:**
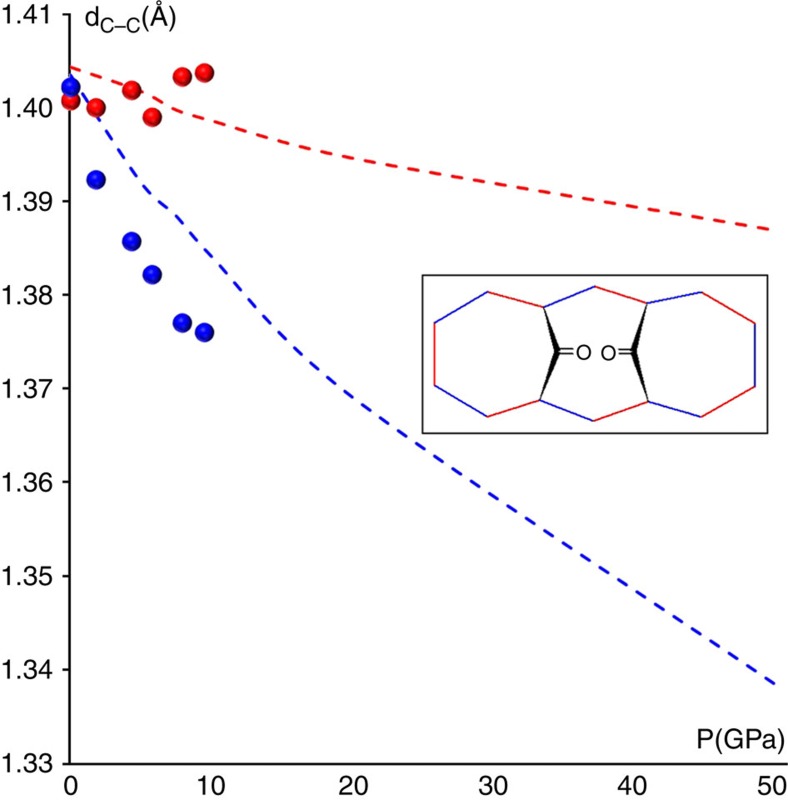
Resonant configurations bond evolution. The average bond distances for hypothetical C–C double bonds of ψ_1_ and ψ_2_ (blue and red, following Fig. 3) as a function of pressure from the single crystal measurements (circles) or the theoretical calculations (dashed lines). Experimental data are corrected for thermal libration effects.

Good indicators of this phenomenon are the average C–C distances of the hypothetical double bonds for each of the two resonant configurations (Figs 3 and 6). At ambient *P*, the observed and calculated pseudo-C_2v_ symmetry implies that the two sets of bonds have coincident average distances (1.396 Å). As *P* increases, however, one of the two resonant configurations of Fig. 3 (ψ1) becomes progressively dominant. This is evident, because all the double bonds of this configuration shorten with respect to the ambient pressure geometry, whereas all the hypothetical double bonds of the alternative configuration (ψ_2_) are unaltered or even slightly elongated (see also Supplementary Fig. 7 and Table 4). From the experimental structure at 9.5 GPa, the average distances of the two sets of bonds are 1.375 and 1.405 Å, respectively. The gap is even larger at 50 GPa, from the theoretical simulations (1.338 vs 1.387 Å, see also Supplementary Fig. 8) This shows that BCA clearly distorts from C_2v_ symmetry, but the C–C bonds in the annulene ring respect one of the two mirror symmetries, namely the C_s_(1) configuration represented in Fig. 2. This distortion destabilizes the molecule (by ca. 65 kcal mol^−1^) not only because of breaking the aromaticity, but also because of other geometrical distortions imposed by the reduced volume available for each molecule, including a significant bending of the bridging CO's, Fig. 1. The distance between them shortens from 2.591 (ambient pressure) to 2.525 Å (at 9.5 GPa, from experiment) or even 2.46 Å (at 50 GPa, from calculations), at the expense of the C=O bonds that experience a small but continuous elongation under pressure. This observation confirms the weak bonding proposed by Destro and Merati^31^, and it suggests a slight strengthening with pressure.

### Electron and current density distribution

The partial localization of one electronic configuration should be visible also from the analysis of the ED distribution. For this reason, we determined the accurate *ρ*(**r**) from the X-ray diffraction intensities measured at 7.7 GPa. We used two methods to reconstruct *ρ*(**r**): the traditional multipolar refinement^22^ and the X-ray constrained wave function^37^. Details of the model refinements are in SupplementaryMethods, whereas the technical details on the necessities and pitfalls of ED determinations at HP will be subject of a forthcoming paper. Here we report only on the most relevant results of the *ρ*(**r**) analysis. To validate the experimental models, the ED was also computed at various pressure points with periodic DFT calculations.

**Figure 7:**
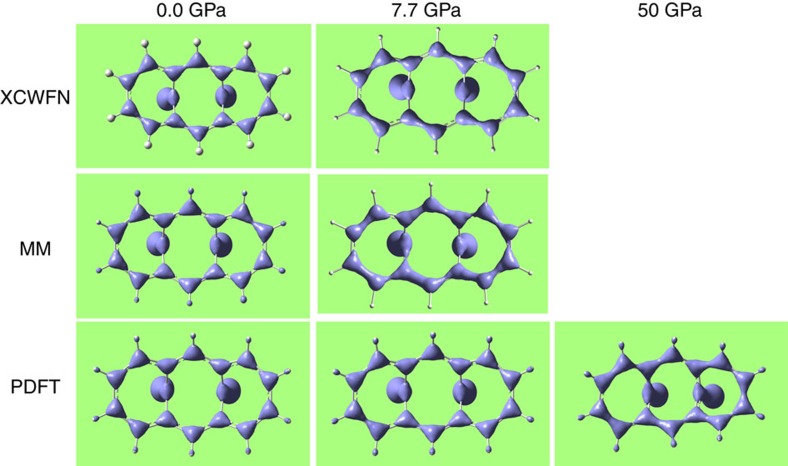
ED distribution of BCA. Plots are shown at various pressure and from various sources: PDFT are periodic DFT calculations at B3LYP level of theory; XCWFN are X-ray constrained wave functions computed at Hartree–Fock level, but constrained against the experimentally measured diffraction intensities; MM is the electron density derived from multipolar expansion, with coefficients refined against experimentally measured intensities. Experimental data at ambient pressure are taken from Destro and Merati^31^, collected at 19 K; the 7.7 GPa data are from this work.

In Fig. 7, we plot *ρ*(**r**) at isosurface values (0.305 arbitrary units) chosen to visualize the ED level of the mixed single–double bonds of the molecular skeleton. At 0.0001 GPa, the theoretical calculations and the experimental model^31^ show an almost perfectly symmetric distribution. The bonds C1–C14 and C2–C3 (and all pseudosymmetry related ones, Fig. 2) display larger amount of ED, in agreement with their shorter distances, whereas C1–C2 and C3–C4 and all pseudosymmetry equivalents, have lower density. This scenario respects the resonance scheme, that, however, partially breaks at 7.7 GPa. In fact, both the experimental models and the periodic DFT calculations demonstrate that bonds C2–C3, C4–C5 and C6–C7 gain ED whereas C1–C2, C3–C4 and C5–C6 loose it (Fig. 7). In the left part of the molecule, the scenario is now closer to a localized configuration because the ED accumulations are clearly associated only with the hypothetical double bonds of ψ_1_ in Fig. 3. On the other hand, in the right part of the molecule a larger delocalization persists. To explain this, one should consider that localizing ψ_1_ implies strengthening bonds C8–C9, C10–C11 and C12–C13 (originally weaker at ambient conditions) at the expense of C7–C8, C9–C10, C11–C12 and C13–C14 (originally stronger). Anyway, C8–C9, C10–C11 and C12–C13 have increased the ED amount compared with their pseudosymmetric counter parts C5–C6, C3–C4 and C1–C2. A complete localization of ψ_1_ would eventually occur at 50 GPa, where only periodic DFT calculations are available without confirmation from the experiment. At this pressure, all the double bonds of ψ_1_ are associated with larger amounts of ED peaks, compared with all single bonds. The molecule has therefore become closer to a cyclic non-aromatic poly-ene.

The qualitative agreement between the experimental models (multipole or X-ray constrained wave function) and the first principle calculations (periodic DFT) at 7.7 GPa is remarkable, which excludes potential biases in the analysis and it allows to thrust the theoretical values computed at 50 GPa.

**Figure 8:**
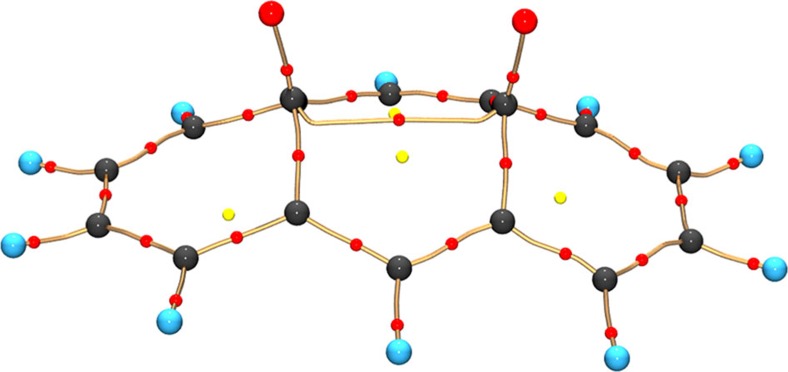
BCA bonding scheme. Molecular graph for BCA at 7.7 GPa from the experimentally refined multipolar model. Atoms are represented by black (carbon), red (oxygen) and blue (hydrogen) spheres, critical points are shown as small red dots. Noteworthy, depending on the refinement model, a bond path interconnecting the two O atoms can also be localized.

From the multipolar model or the X-ray constrained wave function, we could also determine the molecular graph (Fig. 8), that is, the set of lines of maximum ED (bond paths) that interconnect bonded atoms, following QTAIM. The partial localization of one of the two resonant configurations is visible also from the increased ED at the critical points (saddle points along the bond paths). On average, *ρ*(**r**) is 2.29 and 2.15 e Å^−3^ in the partial double or single bonds, respectively, which varies from what observed at ambient pressure at 19 K (on average, 2.05 e Å^−3^ for both configurations).

As observed at the ambient *P* and low *T* by Destro and Merati^31^, a bond path links the two carbonyl carbons, a feature confirmed also by the periodic DFT calculations.

Having an experimental wave function available, we can compute other quantities, otherwise not available from the multipolar model only; in particular, the delocalization indexes (*δ*) (ref. 38) that address the amount of electron pairs shared between two atoms. While the theoretical calculations for the C_2v_ symmetric geometry give on average *δ*_C–C_=1.34e for both configurations, at 7.7 GPa the partially localized double bonds have *δ*_C–C_=1.42e against *δ*_C–C_=1.28e of the partially localized single bonds. For the theoretically calculated structure at 50 GPa, *δ*_C–C_=1.47e and 1.22e, respectively, in keeping with the further shortening of the double bonds and lengthening of the single bonds. Noteworthy, the non-planar and highly strained geometry of BCA would hamper a full localization of double bonds and the ideal *δ*_C–C_=2.0e.

**Figure 9:**
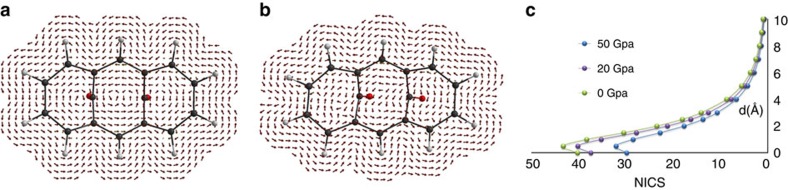
BCA ring current. The calculated current density *J*(**r**) for the molecule of BCA at 0.0001 GPa (**a**) and 50 GPa (**b**). (**c**) NICS scan perpendicularly to the average plane of the BCA ring (anti with respect to the bridging carbonyls).

As anticipated above, the aromaticity of a molecule also affects the current density in the ring and the shielding experienced by the atomic nuclei. Current density can only be calculated, with gauge invariant orbitals for molecules, after geometry optimizations in the crystal at various pressure points. These results are illustrated in Fig. 9, where one can easily see the modified orientation of the current density vectors at 50 GPa. The NICS can be also used to analyse the pressure-induced changes. Recent works suggest to scan the out of plane components along a direction perpendicular to the ring^39^. In BCA, we can scan only along the direction opposed to the two CO bridges, otherwise the shielding of C=O would severely interfere. In agreement with standard aromatic systems, NICS is a bit larger (in absolute value) at *ca.* 0.5–1.0 Å out of the plane, and then it decreases (Fig. 9c). NICS decreases from the ambient pressure to the 50 GPa structure. This is a further proof, based on magnetic criteria, that the aromaticity of BCA decreases as a function of pressure, in keeping with the structural, electronic and energetic criteria above discussed.

## Discussion

We have analysed the pressure-induced loss of aromaticity of a carbonyl annulene. The molecule activates by partially localizing one of the two resonant configurations. This mechanism may occur also in other aromatic systems under pressure and could be representative of the steps that anticipate addition reactions leading to polymerization, like in benzene.

A fortunate circumstance for BCA is that the activation mechanism is induced by a smaller pressure and it does not damage the crystal quality, thus the molecular geometries as well as the ED could be determined with sufficient accuracy to elucidate many important details.

This observation of progressive aromatic loss in BCA is extremely useful to test how different indicators of aromaticity respond to drastic changes of the molecular geometry. Criteria based on C–C distances appear to be extremely sensitive. Moreover, they are quite easy to determine whether single crystal X-ray diffraction is available. Deviations from a uniform distribution of C–C bond distances directly correlate with the raise of molecular energy that can be determined with theoretical methods but not experimentally. While magnetic criteria are also sensitive to an increased or decreased aromaticity, NICS are only available from theoretical calculations, whereas the ^1^H NMR chemical shifts, although measurable even at HP, may not reveal so directly the ongoing changes of electronic configuration. In fact, at each pressure point, the protons would probe the ring current in different positions, due to the geometrical distortions that involve themselves as well. Therefore, they would be unable to reveal the actual modifications of the current density due only to the breaking of aromaticity.

In this study, we have also presented a way to determine experimentally the charge density of a molecular crystal under pressure, which provides additional indicators of the aromaticity based on the topological analysis of the ED. We believe the use of a synchrotron is presently mandatory to achieve the necessary quality and quantity of unique reflections; potential improvements on our setup include the use of poly-nanocrystalline diamonds, which should eliminate the problem of diamond dips (though introducing a significant background), the use of photon counting detectors (possibly with a high Z material such as GaAs), which would reduce the noise and an improved usage of panoramic cells, presently limited in the literature. The ongoing research on new liquid jet microsources of relatively high energy may also enable in the near future such experiments with laboratory sources, nevertheless the present limitations are difficult to overcome and limit the possibilities of such studies, though not their potential. As we have shown for BCA at 7.7 GPa, it was possible to successfully refine a full multipolar model and an X-ray constrained wave function. This enabled us to visualize the partial localization of double and single bonds, which eventually becomes more complete at higher pressure, according to theoretical predictions. ED criteria are seamlessly replicating the geometric criteria that, in this case, provide easily accessible and sufficiently reliable indicators of even minor changes of aromaticity. This cross-validation opens up the possibility of using this method for far more complex observation of ED localization in molecular systems.

## Methods

### HP diffraction measurements

The two separate kinds of experiments were conducted using a Betsa and an own University of Oxford screw driven-type DAC equipped with 0.5 mm culet diamonds. In the first setup a single crystal of BCA was loaded, using a methanol;ethanol 4:1 mixture as pressure medium and ruby fluorescence for pressure measurement. At *P*=7.7 GPa, a second setup was adopted, using two crystals pre-oriented with crystallographic axis almost normal to each. A sufficiently high redundancy was also sought to correct for problems such as diamond dips, which were also separately identified by recording transmission scans through the cell using a diode immediately after the cell itself. With this setup, the overall data redundancy was 4.7 (6.0 for data up to 0.8 Å resolution) and 70% of the unique reflections were measured (88% for data up to 0.8 Å resolution). This guarantees a sufficient sampling of the reciprocal space, especially in the region where the valence electrons are mostly scattering.

For both setups, a monochromatic radiation of 40 keV was focused down to about 90 × 90 μm^2^ and then collimated by pinhole of 30 μm in diameter. In the 7.7 GPa experiment the full beam size was always probing only one of the two crystals, which were separately centred and measured. The beam was notably smaller than the crystal, which means all the beam was effectively used for diffraction by the crystal itself. In the case of beams larger than the crystal, problems may arise from the significant diffraction of diamonds, saturating the detector and of the other elements of the cell, which contribute to background. Data were treated using the dedicated HP routines present in the package of CrysalisPro^40^ for shading areas, carefully assigning a well-describing vector and opening angle to the cell. Diamond reflections were individually masked in a similar way to already described procedures^41^, which proved also important in obtaining a smooth background subtraction from the programme itself. Suspiciously badly fitting reflections were investigated and manually rejected when: (a) their intensity was significantly lower than their equivalents and on the border of masks, (b) their intensity was significantly higher than their equivalents and on the tails of a diamond reflection and (c) they were on images collected at angles were an obvious diamond dip occurred, as revealed by the mentioned transmission scans. No change of space group was detected up to the maximum pressure; therefore, standard refinements were carried on the known structure using ShelXL^42^ as included in the WingX package^43^. The non-standard P2_1_/n space group was used for all determinations for sake of consistency with previous studies^31^.

### Experimental determination and modelling of the ED distribution at 7.7 GPa

To obtain an accurate ED mapping, it is necessary to collect accurately and extensively the X-ray data, up to a sufficient resolution. High resolution is necessary because the large number of parameters of a multipolar model requires many more intensities to match a sufficient observation/parameter ratio. The data set should be sufficiently complete to avoid systematic effects in the refinement. This is particularly cogent for the low angle data, because the valence electrons, which are mainly responsible for aspherical scattering, are not contributing to reflection intensities at higher resolution. To reach the goal of determination of the ED in a crystal, it is also necessary a very accurate measurement of the reflection intensities, which means minimizing the effect of experimental errors such as absorption by the sample, extinction and, very important here, absorption by diamonds and metal gasket. Apart from accurate correction of the data, the modern area detector technologies offer exceptional possibility to improve the precision of the measurement by repeated collection of the same intensities.

The data set collected at 7.7 GPa was an ideal candidate to attempt a determination of accurate ED in BCA, because the pressure is sufficient to reduce the thermal parameters by a factor of *ca.* 4 (making the atomic displacement parameters comparable to those measured at 120 K) and because both crystals were still sufficiently free from damages. Below this pressure, the atomic motion is still too large and above it the hydrostaticity of the medium decreases and therefore some damage could occur to the samples, which does not affect a conventional structure determination but hampers any accurate ED mapping. Data from the two difference crystals were linearly scaled and merged without any weighting scheme using the appropriate routine in WingX.

A full multipolar model could be refined based on the 7.7 GPa data, expanding each C and O atom up to an octupole level and each H atom up to a dipole level (refining only the bond directed dipole). H positions were fixed at values calculated from the periodic DFT calculations. The final R factor is larger than what one could obtain from low temperature experiments at ambient pressure (*ca.* 2% versus 6%), but an accurate analysis of the residuals reveal that they obey a normal distribution and the larger peaks do not occur in important regions of the molecules. This implies that, despite being noisy, the data do not contain systematic errors and the model is therefore not significantly biased. The program XD2006^44^ was used for the multipolar modelling and for the calculation of the ED and the molecular graph. MolCoolQT^45^ and Gaussview^46^ were used for plotting isosurfaces.

### Theoretical calculations

Calculations on molecules (structural optimization, ED, NICS and wing current) were carried out with Gaussian09 (ref. 47), using B3LYP/6-31(2d,2p) level of theory. AimAll^48^ was used to compute and visualize the ED, the theoretical molecular graph and the ring current. Calculations on periodic systems (geometry optimization and ED at various pressure points) were carried out at the same level of theory, including empirical corrections for dispersion effects. The program CRYSTAL14 (ref. 29) was used.

X-ray constrained wave function calculations were carried out using the program TONTO^49^. This method is a calculation of molecular orbitals by means of a modified variational approach, which minimizes a function that couples the Hartree–Fock energy of a molecule and the experimentally observed structure factors of its crystal. The same basis set of previous calculations was used. X-ray constrained wave functions enable to exploit the experimental information, though avoiding a dangerous over-fitting of noisy data that could occur using the multipolar model. This is especially cogent when data are not of the highest quality, such as the X-ray diffraction measured from crystal in a DAC.

## Additional information

**Accession codes:** The X-ray crystallographic coordinates for structures reported in this study have been deposited at the Cambridge Crystallographic Data Centre (CCDC), under deposition numbers: 1438912-1438922. These data can be obtained free of charge from The Cambridge Crystallographic Data Centre via www.ccdc.cam.ac.uk/data_request/cif.

**How to cite this article:** Casati, N. *et al*. Putting pressure on aromaticity along with *in situ* experimental electron density of a molecular crystal. *Nat. Commun.* 7:10901 doi: 10.1038/ncomms10901 (2016).

## Supplementary Material

Supplementary Information Supplementary Figures 1-9, Supplementary Tables 1-6, Supplementary Methods
