# Supplementary material for: Putting pressure on aromaticity along with in situ experimental electron density of a molecular crystal
Source: Nat Commun. 2016 Mar 16;7:10901. doi: 10.1038/ncomms10901 (PMC4799374; doi:10.1038/ncomms10901)
Supplement: Supplementary Information — Supplementary Figures 1-9, Supplementary Tables 1-6, Supplementary Methods [file ncomms10901-s1.pdf]

Supplementary Figures

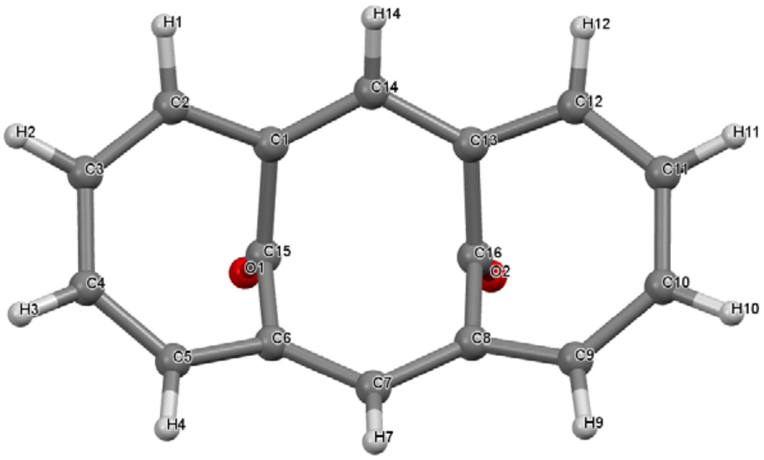

Supplementary Figure 1. The molecular structure of BCA with atomic labelling

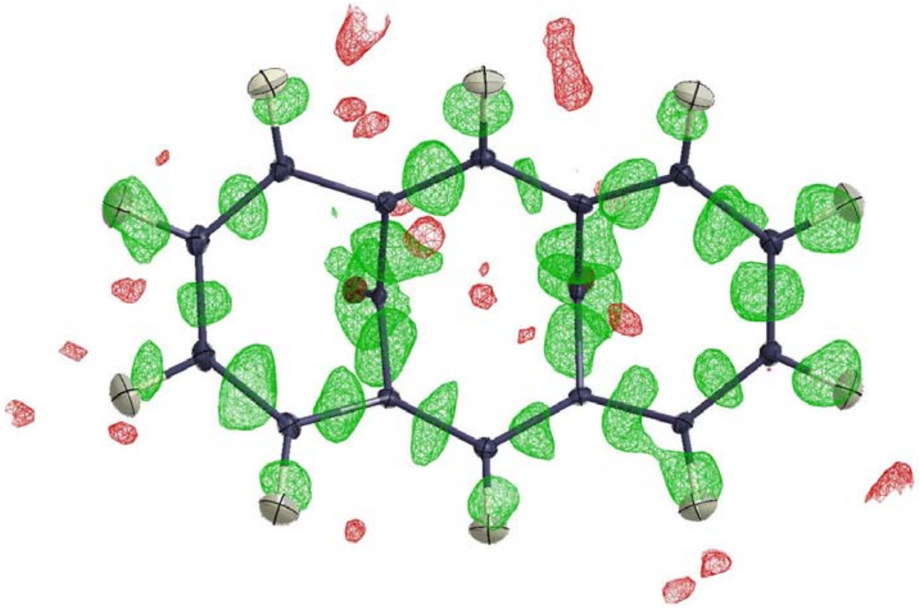

Supplementary Figure 2. The X-X experimental deformation density map, i.e. the Fourier transformation of the difference between observed structure factors and structure factors calculated from high order spherical atom refinement. The map is calculated using low order reflections ($\sin\theta/\lambda < 0.7$ Å^{-1}). Green isosurfaces represent positive values (0.3 eÅ^{-3}) of the deformation densities and they are in fact located in the middle of the bond. Plot drawn with MolCoolQt.[45]

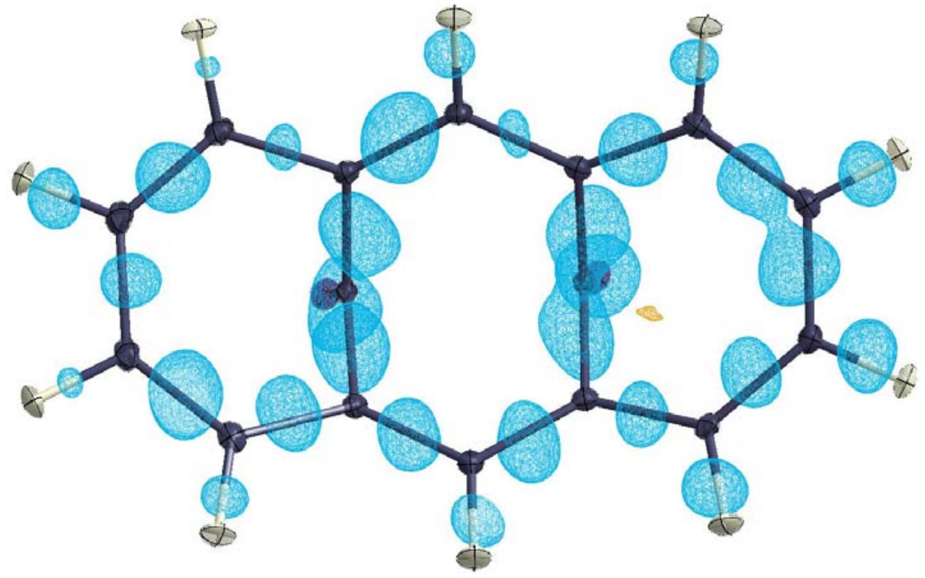

Supplementary Figure 3. The model deformation density map, i.e. the Fourier transformation of the difference between structure factors calculated with the multipole model and structure factors calculated with a spherical atom model. All reflections are used. Blue isosurfaces represent positive values (0.3 eÅ^{-3}) and coincide with those of the experimental deformation density meaning that the multipole model was able to fit all the observed features. Plot drawn with MolCoolQt.[45]

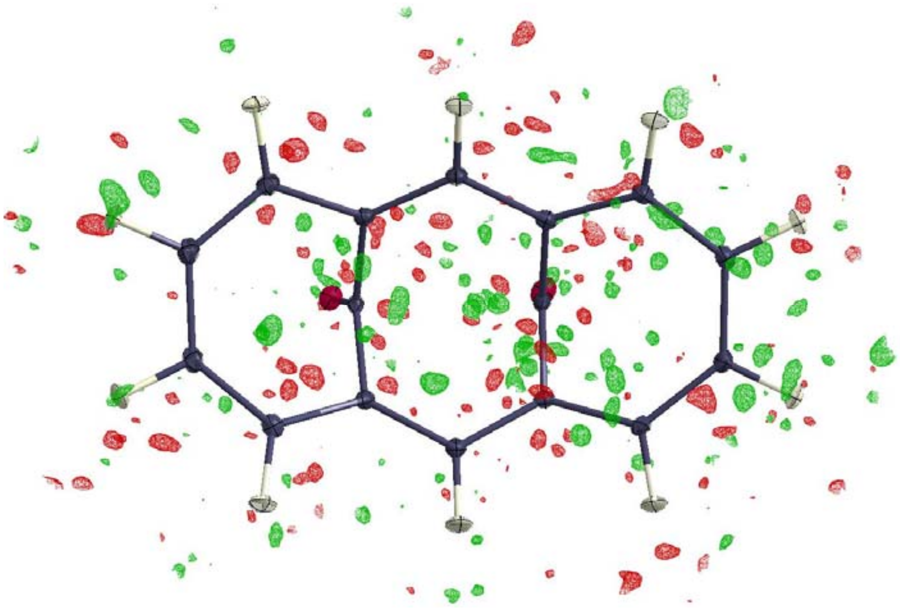

Supplementary Figure 4. The residual deformation density map, i.e. the Fourier transformation of the difference between the observed structure factors and those calculated with the multipole model. All reflections are used. Green isosurfaces represent positive values (+0.3 eÅ^{-3}), red isosurfaces represent negative valued (-0.3 eÅ^{-3}). Plot drawn with MolCoolQt.[45]

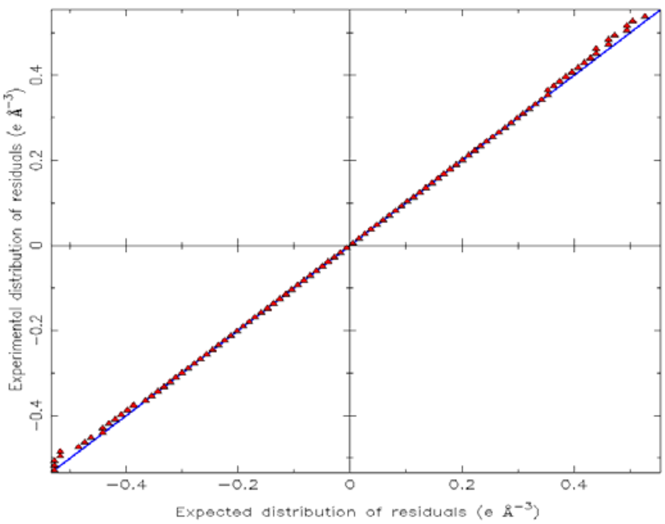

Supplementary Figure 5 The normal probability plot for the distribution of residuals. Plot drawn with XD2006.[44]

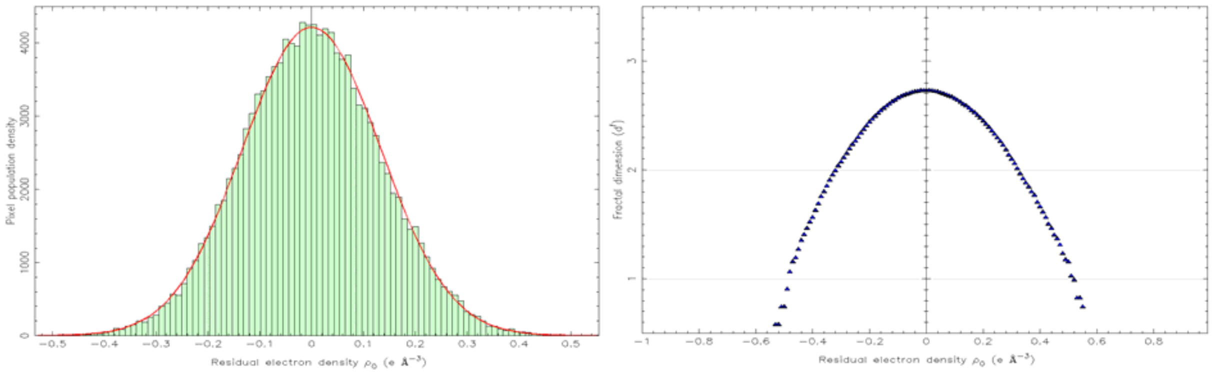

Supplementary Figure 6 Left: pixel population of the residual density in the unit cell after multipolar refinement. The Gaussian shape of this plot indicates that errors do not feature systematic effects and not net residual is found. Right: Fractal dimension of the residual density.[50]

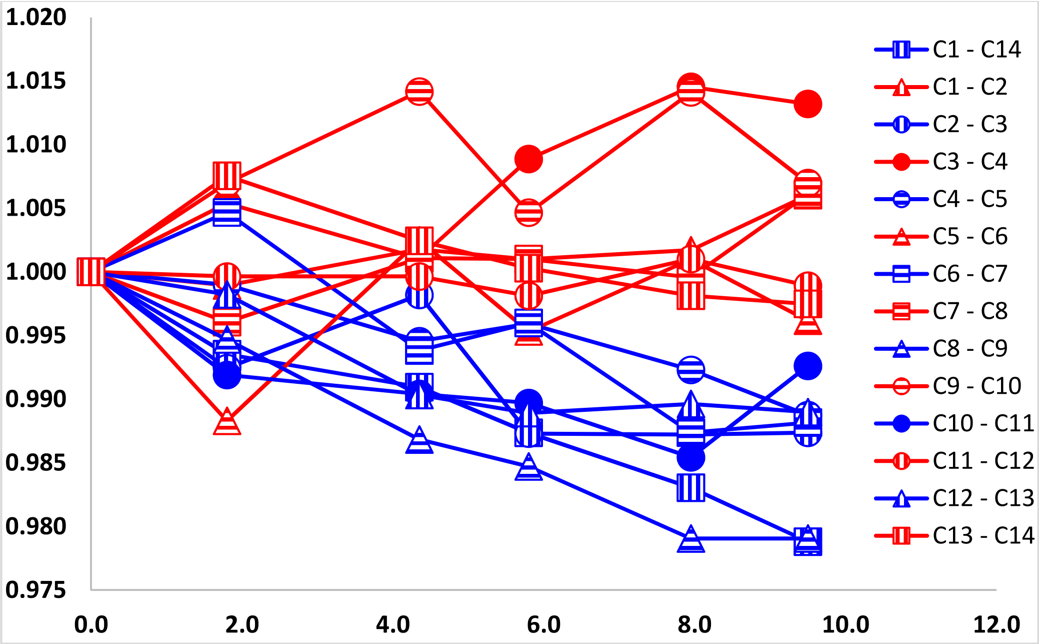

Supplementary Figure 7. Relative compression / expansion of C-C bonds as a function of pressure (in GPa) from experimental modelling). Distances are normalized to the value at ambient pressure and temperature. Red and blue symbols refer to the double bonds of the electronic configurations depicted in Figure 3 of the article. Symbols follow the C_{2v} pseudo-symmetry of the molecule (Figure 2 of the article).

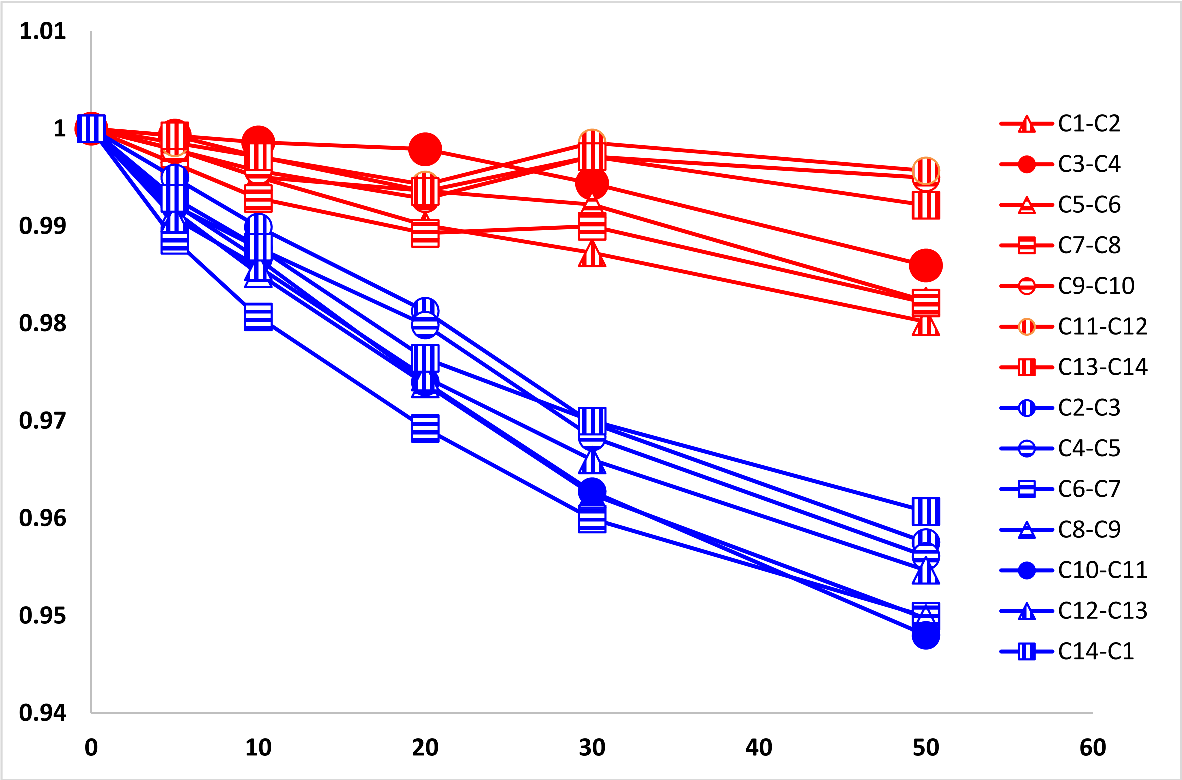

Supplementary Figure 8. Relative compression / expansion of C-C bonds as a function of pressure (in GPA), from periodic DFT calculations. Distances are normalized to the value at ambient pressure and temperature. Red and blue symbols refer to the double bonds of the electronic configurations depicted in Figure 3 of the article. Symbols follow the C_{2v} pseudo-symmetry of the molecule (Figure 2 of the article). Noteworthy, the theoretical simulation is extended up to 50 GPa.

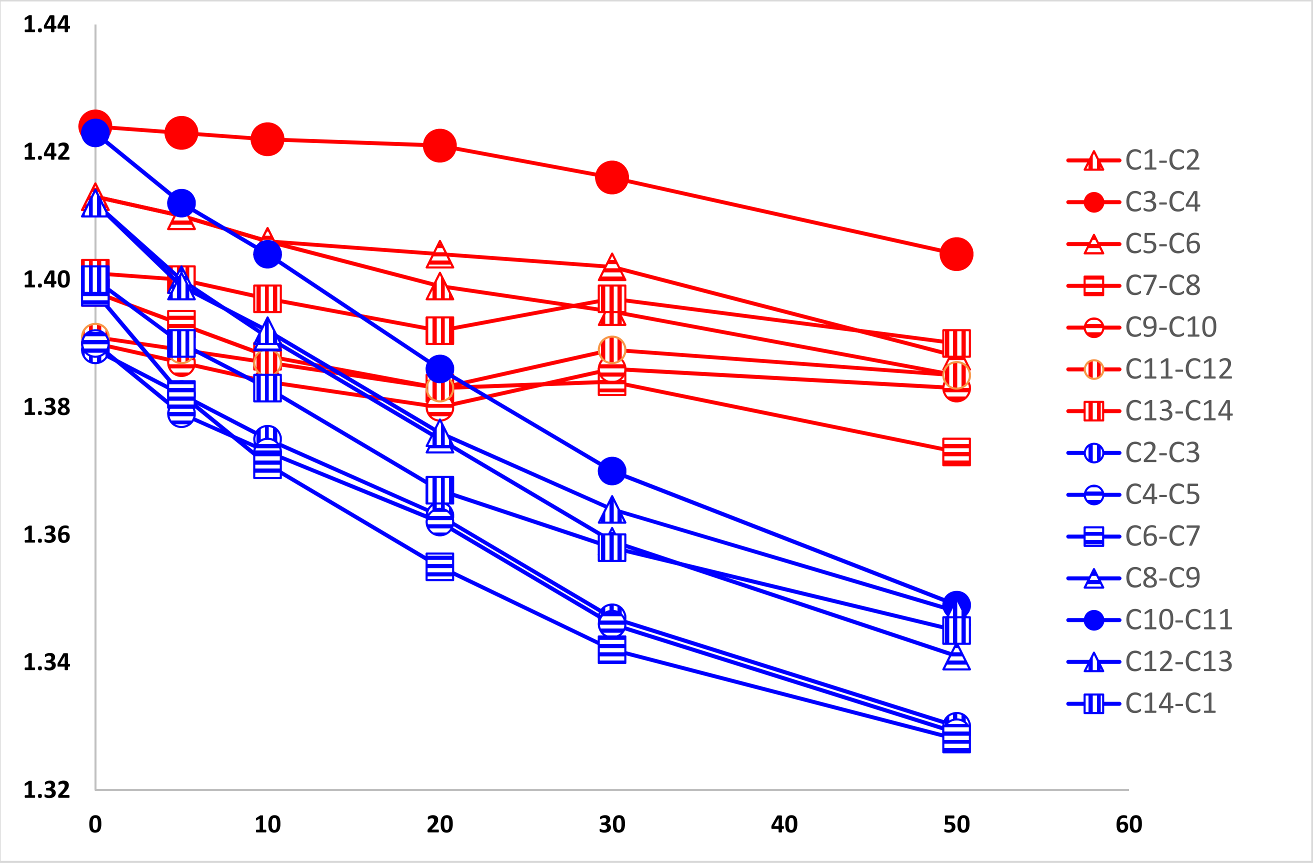

Supplementary Figure 9. The C-C bond distances as a function of pressure (in GPa), from periodic DFT calculations. Red and blue symbols refer to the double bonds of the electronic configurations depicted in Figure 3 of the article. Symbols follow the C_{2v} pseudo-symmetry of the molecule (Figure 2 of the article).

Supplementary Tables

Supplementary Table 1. Selected crystallographic data and spherical atom refinement parameters for the high pressure data collections. The space group $P2_1/n$ remains unchanged during the compression. Resolution of the datasets is 0.8Å

P (GPa)	1.8 GPa	4.35	5.8	7.95	9.5
a (Å)	8.8423(5)	8.7168(4)	8.6563(4)	8.5923(5)	8.5652(5)
b (Å)	12.2033(16)	11.8642(13)	11.7038(12)	11.5463(14)	11.4704(16)
c (Å)	9.1817(12)	8.8601(10)	8.7140(10)	8.5840(10)	8.5109(12)
β (°)	92.780(6)	92.569(5)	92.652(6)	92.826(6)	92.950(8)
V (Å^3)	989.59(19)	915.37(15)	881.88(14)	850.58(15)	835.06(17)
Reflections: Total/unique(>3σ)	5136/1394(992)	7354/1481(1103)	7308/1423(1094)	6584/1367(1012)	3709/1033(712)
R_{int}	0.0683	0.0974	0.0988	0.0851	0.0788
R_1	0.0691	0.0687	0.0635	0.0596	0.0525
Δρ min/max (eÅ^{-3})	-0.340/0.337	-0.441/0.344	-0.310/0.303	-0.318/0.327	-0.211/0.204

Supplementary Table 2. Selected crystallographic data and spherical atom refinement parameters for the low temperature data collections. The space group $P2_1/n$ remains unchanged during the cooling. Resolution is 0.67 Å for all datasets.

T (K)	90	123	183	243	298
a (Å)	9.0425(1)	9.0481(1)	9.6081(1)	9.0759(1)	9.0915(1)
b (Å)	12.6672(1)	12.6786(1)	12.7038(1)	12.7323(1)	12.7606(1)
c (Å)	9.6675(1)	9.6881(1)	9.7292(1)	9.7769(1)	9.8227(1)
β (°)	94.337(1)	94.415(1)	94.501(1)	94.607(1)	94.706(1)
V (Å^3)	1104.11(2)	1108.10(2)	1116.45(2)	1126.14(2)	1135.72(2)
Reflections: Total/unique	13844/3582	14231/3594	14324/3620	14464/3650	14579/3686
R_{int}	0.0252	0.0254	0.0263	0.0286	0.0285
R_1	0.0372	0.0371	0.0384	0.0399	0.0408
Δρ min/max (eÅ^{-3})	-0.24/0.39	-0.22/0.38	-0.21/0.32	-0.19/0.28	-0.16/0.23

Supplementary Table 3. Selected crystallographic data and refinement parameters for the extensive dataset collected at 7.7 GPa and used for the electron density refinement.

a (Å)	8.6187(3)
b (Å)	11.5861(6)
c (Å)	8.6105(3)
β (°)	92.788(3)
V (Å^3)	858.80(6)
Total reflections / unique / observed (I >2σ(I))	23698 / 5047 / 3554
R_{int} (all data)	0.116
R_{int} (I > 2σ(I))	0.079
Number of parameters for the spherical atom refinement[1]	163
R_1 (I > 2σ(I)):	
sinθ/λ < 0.7 Å^{-1}	0.073
sinθ/λ > 0.7 Å^{-1}	0.103
all data	0.089
Δρ min/max (eÅ^{-3}):	
sinθ/λ < 0.7 Å^{-1}	-0.44/0.60
all data	-0.60/0.88
Total number of parameters for the multipolar model[2]	464
R_1 (I > 2σ(I)):	
sinθ/λ < 0.7 Å^{-1}	0.0451
all data	0.0676
Δρ min/max (eÅ^{-3}):	
sinθ/λ < 0.7 Å^{-1}	-0.30/0.29
all data	-0.53/0.55

[1] Using the XD2006 conventions and $\boldsymbol{w_h = 1/\sigma^2(F^2_{h,obs})}$

[2] The refinement was carried out in two steps: 1) high order spherical atom refinement of the position and thermal parameters for C and O atoms (163 parameters); 2) refinement of the multipole coefficients using all reflections (302 parameters). A scale factor was refined in both cases.

Supplementary Table 4. Bond distances from the refined models of the high pressure experiments

P(GPa)	1.8	4.35	5.8	7.7[1]	7.95	9.5
C1 - C14	1.383(5)	1.379(4)	1.374(4)	1.376(2)	1.368(4)	1.362(5)
C1 - C2	1.408(5)	1.412(5)	1.411(4)	1.414(2)	1.412(4)	1.418(5)
C2 - C3	1.366(6)	1.374(5)	1.359(5)	1.373(2)	1.359(4)	1.359(6)
C3 - C4	1.417(6)	1.411(5)	1.422(5)	1.429(3)	1.430(5)	1.428(6)
C4 - C5	1.376(6)	1.370(5)	1.372(4)	1.374(3)	1.367(4)	1.362(5)
C5 - C6	1.393(5)	1.413(5)	1.403(4)	1.413(2)	1.411(4)	1.404(6)
C6 - C7	1.400(5)	1.385(4)	1.388(4)	1.381(4)	1.376(4)	1.377(5)
C7 - C8	1.388(5)	1.395(5)	1.395(4)	1.405(2)	1.393(4)	1.402(5)
C8 - C9	1.405(5)	1.394(4)	1.391(4)	1.387(2)	1.383(4)	1.383(4)
C9 - C10	1.385(6)	1.395(5)	1.382(4)	1.395(2)	1.395(4)	1.385(6)
C10 - C11	1.396(6)	1.394(5)	1.393(5)	1.404(2)	1.387(5)	1.397(6)
C11 - C12	1.383(5)	1.383(5)	1.381(4)	1.396(3)	1.385(4)	1.382(5)
C12 - C13	1.404(5)	1.393(4)	1.391(4)	1.395(2)	1.392(4)	1.391(5)
C13 - C14	1.402(5)	1.395(4)	1.392(4)	1.394(2)	1.389(4)	1.388(5)
C1 - C15	1.467(6)	1.467(5)	1.466(5)	1.470(3)	1.464(5)	1.460(6)
C6 - C15	1.476(5)	1.467(5)	1.476(4)	1.473(2)	1.468(4)	1.475(5)
C8 - C16	1.474(5)	1.471(5)	1.470(4)	1.472(2)	1.464(4)	1.463(5)
C13 - C16	1.469(5)	1.462(5)	1.463(5)	1.473(3)	1.460(5)	1.461(6)
C15 - O1	1.213(4)	1.212(4)	1.214(4)	1.215(2)	1.216(4)	1.223(4)
C16 - O2	1.217(5)	1.221(4)	1.214(4)	1.215(2)	1.223(4)	1.225(5)
C15 - C16	2.557(4)	2.543(4)	2.538(3)	2.539(3)	2.522(3)	2.525(4)

[1] From the multipolar refinement on high resolution data. These data are not directly comparable with those of the other refinements (all based on lower resolution data only), because the thermal motion is here better de-convoluted, giving in general slightly longer C-C distances for all bonds. For sake of homogeneity these data are not included in the plot 7. Anyway the asymmetrical distribution of bonds follows the general trend observed at all pressures.

Supplementary Table 5. Unit cells and coordinates from periodic density functional calculations at various pressures.

0.0 GPa

CELL	9.05941	12.55006	9.68601	90.00000	94.45278	90.00000
C	-0.377641	0.003683	0.280155			
C	-0.277214	-0.071385	0.232106			
C	-0.186268	-0.052337	0.125603			
C	-0.196668	0.029407	0.023385			
C	-0.296959	0.112929	0.005593			
C	-0.389384	0.148614	0.107118			
C	0.472929	0.198323	0.080843			
C	0.367330	0.202274	0.178958			
C	0.214474	0.220574	0.148249			
C	0.102137	0.180252	0.223275			
C	0.107464	0.096172	0.322120			
C	0.226447	0.031813	0.367208			
C	0.375908	0.054634	0.347077			
C	0.488400	-0.020416	0.335581			
C	-0.334794	0.115175	0.249417			
C	0.405666	0.169703	0.324877			
O	-0.250183	0.167934	0.325354			
O	0.447366	0.230590	0.417310			
H	-0.276779	-0.151024	0.275281			
H	-0.108065	-0.114818	0.106357			
H	-0.126813	0.016677	-0.060770			
H	-0.308867	0.151245	-0.094902			
H	0.437897	0.220051	-0.024860			
H	0.181717	0.263122	0.053680			
H	-0.008514	0.206212	0.188935			
H	0.000139	0.071449	0.352075			
H	0.202390	-0.043376	0.415765			
H	0.464559	-0.103262	0.355420			

5.0 GPa

CELL	8.73146	11.79549	8.70066	90.00000	90.40043	90.00000
C	-0.395902	-0.006023	0.291307			
C	-0.277689	-0.081332	0.250295			
C	-0.175206	-0.058387	0.134487			
C	-0.188193	0.023686	0.015161			
C	-0.298984	0.105041	-0.011901			
C	-0.404655	0.144051	0.097821			
C	0.453063	0.192524	0.068049			
C	0.338288	0.194942	0.179602			
C	0.181772	0.209879	0.150517			
C	0.062335	0.168226	0.238675			
C	0.063163	0.077230	0.344121			
C	0.186184	0.008289	0.382884			
C	0.339328	0.038530	0.359808			
C	0.461470	-0.036651	0.345238			
C	-0.355862	0.112469	0.255095			
C	0.370993	0.160513	0.339264			
O	-0.272797	0.170395	0.335576			
O	0.415064	0.224078	0.439976			
H	-0.273127	-0.163100	0.305558			
H	-0.084814	-0.119579	0.118540			
H	-0.109164	0.010880	-0.077502			
H	-0.307207	0.140031	-0.126015			
H	0.421761	0.211974	-0.049003			
H	0.152358	0.251491	0.044442			
H	-0.049936	0.197352	0.205119			
H	-0.048152	0.048264	0.377879			
H	0.163646	-0.075930	0.425675			
H	0.441433	-0.124727	0.365744			

10.0 GPa

CELL	8.62911	11.40846	8.46934	90.00000	92.33038	90.00000
C	-0.389578	-0.009251	0.292646			
C	-0.265882	-0.085007	0.261711			
C	-0.164036	-0.061418	0.145408			
C	-0.182336	0.020202	0.019062			
C	-0.295757	0.102093	-0.015227			
C	-0.402802	0.143706	0.092332			
C	0.453230	0.192290	0.056452			
C	0.340063	0.196804	0.169708			
C	0.181409	0.212041	0.140876			
C	0.064121	0.172558	0.234687			
C	0.067786	0.080301	0.344526			
C	0.191545	0.006554	0.380570			
C	0.344630	0.036836	0.354933			
C	0.467456	-0.040801	0.343521			
C	-0.351716	0.112385	0.254543			
C	0.375196	0.162370	0.333771			
O	-0.265361	0.171830	0.338079			
O	0.414792	0.229395	0.438915			
H	-0.257436	-0.166750	0.324949			
H	-0.069279	-0.121963	0.134629			
H	-0.103836	0.006398	-0.073936			
H	-0.304741	0.136204	-0.133356			
H	0.419874	0.209865	-0.064600			
H	0.147189	0.252503	0.030866			
H	-0.050189	0.203756	0.201604			
H	-0.043308	0.053206	0.383300			
H	0.169276	-0.079690	0.426058			
H	0.447696	-0.130965	0.366895			

20.0 GPa

CELL	8.42203	10.96124	8.14279	90.00000	93.17826	90.00000
C	-0.387361	-0.015765	0.296112			
C	-0.255457	-0.091593	0.276565			
C	-0.151842	-0.066425	0.158502			
C	-0.175192	0.014613	0.023096			
C	-0.292013	0.096630	-0.019424			
C	-0.403969	0.140736	0.086550			
C	0.449738	0.188913	0.044411			
C	0.336384	0.196784	0.162096			
C	0.174875	0.211194	0.134330			
C	0.057606	0.175748	0.236792			
C	0.062287	0.081730	0.350860			
C	0.187174	0.001731	0.380134			
C	0.341665	0.033654	0.353993			
C	0.467287	-0.047062	0.346111			
C	-0.352160	0.109828	0.254982			
C	0.372753	0.163132	0.332085			
O	-0.263917	0.172011	0.342434			
O	0.408616	0.234437	0.441246			
H	-0.241347	-0.172044	0.350644			
H	-0.049177	-0.124223	0.154309			
H	-0.096343	-0.000761	-0.072429			
H	-0.298573	0.130826	-0.142209			
H	0.413855	0.202235	-0.081669			
H	0.136951	0.247733	0.017680			
H	-0.058903	0.210141	0.204621			
H	-0.049643	0.057209	0.395426			
H	0.164322	-0.089083	0.421167			
H	0.447005	-0.138592	0.378077			

30.0 GPa

CELL	7.65375	11.10766	8.19176	90.00000	96.51040	90.00000
C	-0.366960	-0.025723	0.293460			
C	-0.225914	-0.102227	0.271117			
C	-0.128730	-0.081881	0.146847			
C	-0.173952	-0.006231	0.009442			
C	-0.295943	0.080538	-0.022622			
C	-0.396349	0.131922	0.091153			
C	0.448157	0.190934	0.058798			
C	0.337076	0.204321	0.181640			
C	0.159178	0.217608	0.151795			
C	0.034518	0.184345	0.254106			
C	0.042626	0.087317	0.357963			
C	0.176596	0.002738	0.375661			
C	0.345047	0.034026	0.357951			
C	0.477065	-0.049583	0.346275			
C	-0.344222	0.100111	0.258895			
C	0.369876	0.163434	0.349804			
O	-0.302889	0.171065	0.368979			
O	0.366886	0.229687	0.466165			
H	-0.195360	-0.174358	0.355741			
H	-0.014857	-0.136700	0.139893			
H	-0.105384	-0.027455	-0.092144			
H	-0.314488	0.117658	-0.142642			
H	0.394828	0.206410	-0.064506			
H	0.111367	0.245542	0.031955			
H	-0.090878	0.225425	0.225663			
H	-0.074330	0.064550	0.407554			
H	0.147467	-0.089782	0.396378			
H	0.444172	-0.138160	0.374537			

50.0 GPa

CELL	7.27450	10.81346	7.95798	90.00000	98.02326	90.00000
C	-0.355627	-0.031645	0.293900			
C	-0.208621	-0.109723	0.274250			
C	-0.113330	-0.089076	0.146186			
C	-0.168970	-0.013532	0.004688			
C	-0.295315	0.075355	-0.028315			
C	-0.395620	0.129076	0.088111			
C	0.442934	0.190953	0.056389			
C	0.334541	0.208968	0.184973			
C	0.148059	0.217558	0.153626			
C	0.020272	0.186281	0.260242			
C	0.031831	0.086440	0.362826			
C	0.172109	-0.000746	0.373379			
C	0.346791	0.033382	0.360485			
C	0.482323	-0.053057	0.347188			
C	-0.340963	0.096710	0.259085			
C	0.368738	0.165451	0.355963			
O	-0.323039	0.169140	0.377028			
O	0.347748	0.230546	0.474472			
H	-0.169396	-0.179658	0.365540			
H	0.008050	-0.142258	0.139659			
H	-0.101346	-0.035547	-0.100044			
H	-0.315043	0.114730	-0.150355			
H	0.378639	0.203661	-0.069139			
H	0.094417	0.238208	0.028258			
H	-0.108524	0.232667	0.234425			
H	-0.084697	0.064839	0.421012			
H	0.144219	-0.096497	0.385377			
H	0.442935	-0.142508	0.375080			

Supplementary Table 6 Results of the X-ray constrained wave function calculations.

R_1 ($I > 2\sigma(I)$):	
$\sin\theta/\lambda < 0.7$ Å^{-1}	0.055
all data	0.066
χ^2 ($I > 2\sigma(I)$):	
$\sin\theta/\lambda < 0.7$ Å^{-1}	1.42
all data	2.33

Supplementary Methods

Low Temperature data collections

Single crystal X-ray diffraction data were collected at several temperatures, up to a resolution of 0.67Å, using an Agilent SuperNova diffractometer, equipped with Mo Kα microsource X-ray tube, with Al filter and mirror optics. An Oxford Cryosystem Cryostream 700 was used for cooling the crystals. The most relevant crystallographic parameters of these data collections are summarized in Supplementary Table 2.

Remark: X-ray diffraction at variable temperature did not reveal any anomalous thermal motion, which indicates that no disorder between two localized configurations occurs. The arguments proposed by Bürgi[51] to dismiss the potential disorder of two configurations in solid state form of benzene, would not be necessary here. In fact, a hypothetical disorder between two localized configurations of BCA would imply quite large distances between disordered atomic positions, very likely resolvable by a simple refinement against the X-ray data or otherwise very much visible in anomalously large atomic displacement parameters, if only an "average" configuration is refined. In addition, the calculated structure in the crystal (which is inherently ordered) is in almost perfect agreement with the experimental model.

Multipolar refinement of the data at 7.7 GPa

The multipole refinement was performed using the XD2006 program package[52] which adopts the Hansen-Coppens formalism[53] to model the electron density using atom-centred multipoles:

$$\rho_{atom}(\boldsymbol{r}) = \rho_{core}(\boldsymbol{r}) + P_v\,\kappa\,\rho_{valence}(\kappa\,\boldsymbol{r}) + \Delta\rho(\boldsymbol{r}) \qquad (1)$$

where

$$\Delta\rho(\boldsymbol{r}) = \sum_{l=0}^{l_{max}} \kappa_l'\; R_l(\kappa_l' r)\; \sum_{m=-l}^{+l} P_{lm}\; Y_{lm}(\theta,\varphi) \qquad (2)$$

The core and spherical valence density are computed from Slater-type orbitals using the zero order regular approximation level of theory.[52] Single-zeta orbitals with energy-optimized Slater exponents are used for the radial part of the deformation terms.[54] κ and κ_l' are parameters that enable contraction or expansion of the density shells. P_v and P_{lm} represent the population parameters for the valence and deformation density multipoles, respectively. The angular functions Y_{lm} are density normalized real spherical harmonics. The function minimized in the least-squares procedure was $\sum_{\boldsymbol{h}} w_{\boldsymbol{h}} \left(F_{\boldsymbol{h},obs} - \eta\, F_{\boldsymbol{h},calc}\right)^2$ using weight of $w_{\boldsymbol{h}} = 1/\sigma^2\left(F_{\boldsymbol{h},obs}^2\right)$. Reflections with $I_{\boldsymbol{h},obs} > 2\sigma_{\boldsymbol{h},obs}\left(I_{\boldsymbol{h},obs}\right)$ were included in the refinement. Given the small crystal samples and the very short wavelength, no extinction correction was necessary. The multipole expansion was truncated at the octupolar level (l_{max} = 3) for C and O atoms. For H atoms, only bond-directed dipoles and quadrupoles were applied. This was possible because positions of H atoms were fixed using periodic DFT calculated C-H distances at 7.5 GPa and atomic anisotropic displacement parameters (ADP) u_{ij} were calculated based on C and O ADP's with the software SHADE[55] and no longer refined.

Several refinement strategies were tested using the above described model parameters. Because of the lower data completeness, it was found more appropriate refining the atomic positions and ADPs of C and O atoms with high angle data (sinθ/λ > 0.7 Å^{-1}; 163 parameters), then calculating the ADP's for Hydrogen atoms and then keeping the position and displacement parameters fixed for the refinement of all the multipole coefficients (302 variables, reflection/parameters = 11.8). H atoms correlated by pseudo-symmetry (see Figure 2 of the article) were constrained to have the same set of multipole coefficients. κ and κ_l' could be independently refined for C and O atoms, using the same

κ_l' for dipoles, quadrupoles and octupoles. For H atoms, κ and κ_l' were fixed at the standard value of 1.2. This refinement produced correlation coefficients below 0.7 for all pairs of variables but for κ and P_v of O(1) and O(2) (0.82 in both cases).

Noteworthy, the preliminary high order refinement gave a quite satisfactory Hirshfeld rigid bond test,[56] with only four bonds exceeding the limit of 1.0 10^{-3} Å^2 amplitude difference with a maximum of 1.4 10^{-3} Å^2 for the two C=O bonds. Because the atomic position and thermal parameters were fixed during the following refinement steps, the Hirshfeld test remain identical. On the contrary, if ADPs and atomic coordinates are simultaneously refined with multipole parameters (464 variables overall, reflection/parameters = 7.7), the Hirshfeld rigid bond test is much less satisfactory (6 bonds exceed the limit with differences up to 3.0 10^{-3} Å^2), caused by a large correlation between u_{ij} and κ parameters, without much improvement of the agreement indices. For these reasons, the model constructed with high order refinement of positions and thermal parameters and subsequent refinement of multipoles was judged to be the more adequate. Noteworthy, this procedure is always recommended to better de-convolute electron density from thermal motion (see also Dos Santos *et al.*[57]).

Wave function refinement

X-ray constrained wave function calculations were carried out using the program TONTO,[58] following the procedure originally introduced by Jayatilaka and Grimwood.[59] The input geometry and thermal parameters were those obtained from the multipolar refinement. The molecular orbital calculations were carried out at Hartree Fock level, using a constraint to the experiment up to λ_{max} = 1, after verifying that no significant improvement in χ^2 could be obtained. The wave function file obtained with this calculation was then used to compute the electron density features reported in the article, using the program AimAll.[60]

Supplementary References

[50] K. Meindl, J. Henn " Foundations of residual-density analysis" *Acta Cryst.*, **A64**, 404-418 (2008)

[51] H. B. Bürgi "Getting More out of Crystal-Structure Analyses", *Helvetica Chimica Acta*, **86**, 1625–1640 (2003).

[52] A. Volkov, P. Macchi, L. J. Farrugia, C. Gatti, P. Mallinson, T. Richter, T. Koritsanszky, XD2006 - A Computer Program Package for Multipole Refinement, Topological Analysis of Charge Densities and Evaluation of Intermolecular Energies from Experimental and Theoretical Structure Factors. 2006.

[53] N. K. Hansen, P. Coppens "Testing aspherical atom refinements on small-molecule data sets." *Acta Cryst.*, **A34**, 909–921 (1978).

[54] E. Clementi, D. L. Raimondi, "Atomic screening constants from SCF functions." *J. Chem. Phys.*, **38**, 2686-2689 (1963).

[55] A.O. Madsen, "SHADE: web server for estimation of hydrogen anisotropic displacement parameters." *J. Appl. Cryst.*, **39**, 757-758. (2006).

[56] F. L. Hirshfeld, "Can X-ray data distinguish bonding effects from vibrational smearing?" *Acta Cryst.*, **A32**, 239-244 (1976).

[57] L. H. R. Dos Santos, A. Genoni, P. Macchi, "Unconstrained and X-ray constrained Extremely Localized Molecular Orbitals: analysis of the reconstructed electron density" *Acta Cryst. Sec. A*, **A70**, 532-551 (2014)

[58] D. Jayatilaka, D. J. Grimwood "Tonto: A Fortran Based Object-Oriented System for Quantum Chemistry and Crystallography" *Lecture Notes in Computer Science*, *2660*, 142-151 (2003).

[59] D. Jayatilaka, D. J. Grimwood "Wavefunctions derived from experiment. I. Motivation and theory." *Acta Cryst.*, **A57**, 76-86 (2001).

[60] AIMAll (Version 14.11.23), Todd A. Keith, TK Gristmill Software, Overland Park KS, USA (2014) (aim.tkgristmill.com).
